# Sex and gender differences in the use of oral anticoagulants for non-valvular atrial fibrillation: A population-based cohort study in primary health care in catalonia

**DOI:** 10.3389/fphar.2023.1110036

**Published:** 2023-02-07

**Authors:** Maria Giner-Soriano, Oriol Prat-Vallverdú, Dan Ouchi, Carles Vilaplana-Carnerero, Rosa Morros

**Affiliations:** ^1^ Fundació Institut Universitari Per a la Recerca a l’Atenció Primària De Salut Jordi Gol i Gurina (IDIAPJGol), Barcelona, Spain; ^2^ Universitat Autònoma De Barcelona (Cerdanyola del Vallès), Bellaterra, Spain; ^3^ Marketing Farmacéutico and Investigación Clínica, Barcelona, Spain; ^4^ Plataforma SCReN, UIC IDIAPJGol, Barcelona, Spain; ^5^ Departament De Farmacologia, Terapèutica i Toxicologia, Universitat Autònoma De Barcelona (Cerdanyola del Vallès), Bellaterra, Spain; ^6^ Institut Català De la Salut, Barcelona, Spain

**Keywords:** oral anticoagulants (OACs), atrial fibrillation, gender differences, adherence–compliance–persistance, electronic health records–EHR, primary health care (PHC)

## Abstract

**Objectives:** To describe the sex and gender differences in the treatment initiation and in the socio-demographic and clinical characteristics of all patients initiating an oral anticoagulant (OAC), and the sex and gender differences in prescribed doses and adherence and persistence to the treatment of those receiving direct oral anticoagulants (DOAC).

**Material and methods:** Cohort study including patients with non-valvular atrial fibrillation (NVAF) who initiated OAC in 2011–2020. Data proceed from SIDIAP, Information System for Research in Primary Care, in Catalonia, Spain.

**Results:** 123,250 people initiated OAC, 46.9% women and 53.1% men. Women were older and the clinical characteristics differed between genders. Women had higher risk of stroke than men at baseline, were more frequently underdosed with DOAC and discontinued the DOAC less frequently than men.

**Conclusion:** We described the dose adequacy of patients receiving DOAC, finding a high frequency of underdosing, and significantly higher in women in comparison with men. Adherence was generally high, only with higher levels in women for rivaroxaban. Persistence during the first year of treatment was also high in general, being significantly more persistent women than men in the case of dabigatran and edoxaban. Dose inadequacy, lack of adherence and of persistence can result in less effective and safe treatments. It is necessary to conduct studies analysing sex and gender differences in health and disease.

## Introduction

Atrial fibrillation (AF) is the most common form of chronic arrhythmia, affecting 2–3% of the population in Europe and USA (Kirchhof, 2017; Hindricks et al., 2021) or 4.4% of the Spanish population older than 40 (Gómez-Doblas et al., 2014). It is associated with several cardiovascular conditions, and it increases the risk of stroke. Men are more often affected by AF, although women have a higher risk of experiencing stroke (Lip et al., 2012; Hindricks et al., 2021). To prevent stroke in non-valvular atrial fibrillation (NVAF), oral anticoagulants (OAC) are usually prescribed; vitamin K antagonists (VKA) have been used for years and since 2011 in Spain (Agencia Española de Medicamentos y Productos Sanitarios, 2012) direct oral anticoagulants (DOAC) were introduced.

Several authors have described the trends of use of OAC for stroke prevention in NVAF in the recent years (Basaran et al., 2017; Wilkinson et al., 2021; Calderon et al., 2022). From 2014 to 2017, there were more patients with NVAF initiating OAC in our setting (from 67.9% to 81.9%), with an increase of DOAC over time (Programa d’harmonització farmacoterapèutica et al., 2019; Giner-Soriano et al., 2020).

Also diverse studies, including some conducted by our group (Giner-Soriano et al., 2016; 2020; Gomez-Lumbreras et al., 2018), have described the clinical profile of OAC-users (Giner-Soriano et al., 2016; Rodríguez-Mañero et al., 2017), for instance Rodríguez-Mañero et al. described different clinical characteristics and outcomes in octogenarians with AF to those in younger people; the adequacy of the prescription (Dalmau Llorca et al., 2021), reporting inadequate OAC prescriptions in up to 67.6% of the cases; or the adherence and persistence to the treatment, using different methods and finding different compliance levels (Hurtado-Navarro et al., 2018; Banerjee et al., 2020; Sabaté et al., 2021).

Nevertheless, not many studies have described all these aspects highlighting sex and gender differences (Dagres et al., 2007; Avgil Tsadok et al., 2015; Loikas et al., 2017; Tamargo et al., 2017; Raccah et al., 2018), even knowing that women are less likely to receive OAC despite their higher risk of stroke in comparison with men, as they may respond differently to cardiovascular medications, have different incidence of adverse events, or may require different VKA doses (Raccah et al., 2018).

Differences in dosage in women and men have not been analysed either, even taking into account that dose reductions are required for all DOAC depending on different clinical conditions (See Supplementary file, Supplementary Table S1) (European Medicines Agency, 2012b; European Medicines Agency, 2013c; European Medicines Agency, 2016a; Boehringher Ingelheim International GmbH. London (United Kingdom): European Medicines Agency (EMA); 2008, 2013), that under- and overdosing may impact on the effectiveness and safety of these drugs (Shen et al., 2021), that dose adequation has been scarcely described (Programa d’harmonització farmacoterapèutica. et al., 2019) and that sex and gender analyses need to be routinely conducted in research to help decision-making therapeutic process, harm reduction and health equality promotion (Solans-Domènech et al., 2022), taking into account that “sex” refers to biological aspects, such as pharmacokinetic, pharmacodynamic and clinical differences of AF and OAC treatment; and “gender” refers to the sociocultural ones, as how their stroke risk is perceived by the prescribers and how this impacts on OAC prescription (Madsen et al., 2017; Raccah et al., 2018).

In this context, we conducted a study on the use, effectiveness, and safety of OAC prescribed for stroke prevention in NVAF from 2011–2020 in a Primary Health Care (PHC) cohort in Catalonia, Spain. The objectives of the present manuscript were: 1) To describe sex and gender differences in the treatment initiation and in the socio-demographic and clinical profile for all patients who initiated an OAC prescription; and 2) To describe sex and gender differences in the prescribed doses and the adherence and persistence for those initiating DOAC.

## Material and methods

### Study design

Population-based cohort study including adults with NVAF who initiated OAC treatment.

### Population included

We included all ≥18 years-old individuals with an active diagnosis of NVAF registered in PHC electronic records who initiated treatment with OAC from January 2011 to December 2020. Patients were followed-up from the day of initiation of treatment with OAC up to death, disenrollment from the database, end of treatment or end of study period.

### Population excluded

We excluded people with valvular AF and people using DOAC for indications other than NVAF: those diagnosed with pulmonary embolism or deep vein thrombosis during the previous 12 months to the OAC initiation, and those receiving OAC for surgical prophylaxis of hip or knee replacement during the previous 6 months.

### Data source

The data source is the Information System for Research in Primary Care (SIDIAP) (Recalde et al., 2022; SIDIAP, 2022), which captures clinical information of approximately 5,8 million people from Catalonia, Spain (around 80% of the Catalan population). This information is pseudonymized, originated from different data sources: 1) ECAP (electronic health records in PHC in Catalonia); including socio-demographic characteristics, residents in nursing homes/long-term care facilities (LTCF), comorbidities registered as International Classification of Diseases (ICD)-10 codes (see Supplementary file, Table 1) (WHO, 2019), specialist referrals, clinical parameters, toxic habits, sickness leave, date of death, laboratory test data, and drug prescriptions issued in PHC, registered as Anatomical, Therapeutic, Chemical classification system (ATC) codes (see Supplementary file, Table 2) (WHO Collaborating Centre for Drug Statistics Methodology, 2022); 2) pharmacy invoice data corresponding to the PHC drug prescriptions, which includes the number of packages dispensed each month; and 3) database of diagnoses at hospital discharge (CMBD) (CatSalut. Servei Català de la Salut, 2022).

**TABLE 1:** Population with non-valvular atrial fibrillation initiating treatment with oral anticoagulants in 2011–2020.

Overall N = 123,250	ApixabanN = 16,310 (13.2%)	DabigatranN = 10,150 (8.2%)	EdoxabanN = 13,990 (11.4%)	RivaroxabanN = 5,891 (4.8%)	AcenocumarolN = 72,797 (59.1%)	WarfarinN = 4,112 (3.3%)
New users, N= 116,334 (94.4%)^a^	13,866 (85.0%)	8,655 (85.3%)	12,045 (86.1%)	5,249 (89.1%)	72,726 (99.9%)	3,793 (92.2%)
Non-naïve, N= 6,916 (5.6%)^a^	244 (15.0%)	1,495 (14.7%)	1,945 (13.9%)	642 (10.9%)	71 (0.1%)	319 (7.8%)
Women N= 57,832 (46.9%)^a^	7,976 (13.8%)	4,255 (7.4%)	6,192 (10.7%)	2,816 (4.9%)	34,725 (60.0%)	1,868 (3.2%)
Men N= 65,418 (53.1%)^a^	8,334 (12.7%)	5,895 (9.0%)	7,798 (11.9%)	3,075 (4.7%)	38,072 (58.2%)	2,244 (3.4%)

OAC, oral anticoagulants.

^a^ Column percentages.

**TABLE 2:** Baseline sociodemographic and clinical characteristics of patients included in the study.

N (%)	Overall OACN = 123,250			DOACN = 46,341 (37.6)			VKAN = 77,269 (62.4)		
—	Women N = 57,832 (46.9)	Men N = 65,418 (53.1)	*p*-value	WomenN = 21,239 (45.8)	Men N = 25,102 (54.2)	*p*-value	WomenN = 36,953 (47.8)	MenN = 40,316 (52.2)	*p*-value
Mean age (SD)	78.0 (9.5)	73.4 (11.1)	<0.001	78.2 (10.4)	72.5 (12.4)	<0.001	77.9 (8.9)	74.0 (10.2)	<0.001
≥80	28,715 (49.7)	21,446 (32.8)	—	10,982 (51.7)	8,217 (32.7)		17,733 (48.5)	13,229 (32.8)	
Smoking	—	—	<0.001	—	—	<0.001	—	—	<0.001
*Missing*	1,294 (2.2)	1,295 (2.0)		482 (2.3)	686 (2.7)		812 (2.2)	609 (1.5)	
*Yes*	3,571 (6.2)	14,301 (21.9)		1,268 (6.0)	4,816 (19.2)		2,303 (6.3)	9,485 (23.5)	
*Ex-smoker*	8,062 (13.9)	28,000 (42.8)		3,360 (15.8)	11,149 (44.4)		4,702 (12.8)	16,851 (41.8)	
*No*	44,905 (77.6)	21,822 (33.4)		16,129 (75.9)	8,451 (33.7)		28,776 (78.6)	13,371 (33.2)	
Alcohol intake	—	—	<0.001	—	—	<0.001	—	—	<0.001
*Missing*	9,450 (16.3)	10,343 (15.8)		4,082 (19.2)	5,068 (20.2)		5,368 (14.7)	5,275 (13.1)	
*Risk consumption*	282 (0.5)	2,416 (3.7)		99 (0.5)	805 (3.2)		183 (0.5)	1,611 (4.0)	
Mean BMI, kg/m^2^ (SD)	30.0 (5.9)	29.1 (4.8)	<0.001	29.6 (5.9)	28.9 (4.8)	<0.001	30.2 (5.9)	29.3 (4.8)	<0.001
*Missing*	13,064 (22.6)	13,634 (20.8)	<0.001	5,466 (25.7)	6,258 (24.9)	<0.001	7,598 (20.8)	7,376 (18.3)	<0.001
*≥*30 kg*/m* ^*2*^	20,191 (34.9)	19,605 (30.0)	—	6,622 (31.2)	6,802 (27.1)	—	13,569 (37.1)	12,803 (31.8)	—
Mean GFR, mL/min/1,73m^2^ (SD)	71.8 (24.9)	76.6 (25.0)	<0.001	72.5 (25.3)	77.5 (23.8)	<0.001	71.4 (24.6)	76.0 (25.7)	<0.001
*Missing*	5,201 (9.0)	6,437 (9.8)	—	2,042 (9.6)	2,942 (11.7)	—	3,159 (8.6)	3,495 (8.7)	—
<30	1,460 (2.5)	1,349 (2.1)	<0.001	428 (2.0)	300 (1.2)	<0.001	1,032 (2.8)	1,049 (2.6)	<0.001
30–44	5,006 (8.7)	3,684 (5.6)	—	1,880 (8.9)	1,348 (5.4)	—	3,126 (8.5)	2,336 (5.8)	—
45–59	10,443 (18.1)	8,714 (13.3)	—	3,744 (17.6)	3,297 (13.1)	—	6,699 (18.3)	5,417 (13.4)	—
≥60	35,722 (61.8)	45,234 (69.1)	—	13,145 (61.9)	17,215 (68.6)	—	22,577 (61.7)	28,019 (69.5)	—
HAS-BLED	—	—	<0.001	—	—	<0.001	—	—	<0.001
Mean (SD)	2.1 (1.0)	2.2 (0.96)	—	2.0 (0.93)	1.8 (1.1)	—	2.4 (0.95)	2.2 (1.0)	—
0	1,693 (2.9)	4,465 (6.8)	—	1,085 (5.1)	2,908 (11.6)	—	608 (1.7)	1,557 (3.9)	—
1	9,973 (17.2)	14,265 (21.8)	—	4,344 (20.5)	6,340 (25.3)	—	5,629 (15.4)	7,925 (19.7)	—
2	25,051 (43.3)	25,781 (39.4)	—	9,890 (46.6)	9,916 (39.5)	—	15,161 (41.4)	15,865 (39.4)	—
3	16,089 (27.8)	15,381 (23.5)	—	4,802 (22.6)	4,689 (18.7)	—	11,287 (30.8)	10,692 (26.5)	—
≥4	5,026 (8.7)	5,526 (8.4)	—	1,118 (5.3)	1,249 (5.0)	—	3,908 (10.7)	4,277 (10.6)	—
Comorbidities									
≥1	52,034 (90.0)	57,470 (87.9)	<0.001	19,102 (89.9)	21,679 (86.4)	<0.001	32,932 (89.1)	35,791 (88.8)	<0.001
≥3	23,835 (41.2)	28,584 (43.7)	<0.001	9,349 (44.0)	11,180 (44.5)	<0.001	14,486 (39.2)	17,404 (43.2)	<0.001
Cancer	7,873 (13.6)	12,685 (19.4)	<0.001	3,352 (15.8)	5,340 (21.3)	<0.001	4,521 (12.4)	7,345 (18.2)	<0.001
Diabetes	14,326 (24.8)	19,741 (30.2)	<0.001	5,209 (24.5)	7,304 (29.1)	<0.001	9,117 (24.9)	12,437 (30.8)	<0.001
Dyslipidaemia	27,948 (48.3)	28,338 (43.3)	<0.001	10,460 (49.2)	10,930 (43.5)	<0.001	17,488 (47.8)	17,408 (43.2)	<0.001
Hypertension	43,481 (75.2)	44,397 (67.9)	<0.001	15,697 (73.9)	16,490 (65.7)	<0.001	27,784 (75.9)	27,907 (69.2)	<0.001
Ischaemic heart disease	5,621 (9.7)	12,556 (19.2)	<0.001	2,243 (10.6)	5,017 (20.0)	<0.001	3,378 (9.2)	7,539 (18.7)	<0.001
Previous stroke	3,452 (6.0)	4,158 (6.4)	0.005	1,578 (7.4)	1,837 (7.3)	0.660	1,874 (5.1)	2,321 (5.8)	<0.001
Comedications									
ACEI	18,162 (31.4)	23,918 (36.6)	<0.001	6,216 (29.3)	8,419 (33.5)	<0.001	11,946 (32.6)	15,499 (38.4)	<0.001
Antiarrhythmic agents and digoxin	15,551 (26.9)	15,296 (23.4)	<0.001	6,026 (28.4)	6,540 (26.1)	<0.001	9,525 (26.0)	8,756 (21.7)	<0.001
Antidiabetic agents	12,342 (21.3)	17,437 (26.7)	<0.001	4,436 (20.9)	6,493 (25.9)	<0.001	7,906 (21.6)	10,944 (27.1)	<0.001
Antiplatelets	7,825 (13.5)	13,275 (20.3)	<0.001	2,742 (12.9)	4,645 (18.5)	<0.001	5,083 (13.9)	8,630 (21.4)	<0.001
ARB	15,646 (27.1)	14,961 (22.9)	<0.001	5,918 (27.9)	5,969 (23.8)	<0.001	9,728 (26.6)	8,992 (22.3)	<0.001
Beta-blockers	29,708 (51.4)	32,568 (49.8)	<0.001	11,868 (55.9)	13,629 (54.3)	0.001	17,840 (48.8)	18,939 (47.0)	<0.001
Calcium channel blockers	12,585 (21.8)	15,093 (23.1)	<0.001	4,485 (21.1)	5,422 (21.6)	0.210	8,100 (22.1)	9,671 (24.0)	<0.001
NSAID	7,696 (13.3)	5,406 (8.3)	<0.001	3,106 (14.6)	2,221 (8.8)	<0.001	4,590 (12.5)	3,185 (7.9)	<0.001
Proton pump inhibitors	33,389 (57.7)	33,028 (50.5)	<0.001	12,501 (58.9)	12,627 (50.3)	<0.001	20,888 (57.1)	20,401 (50.6)	<0.001
Statins	23,189 (40.1)	31,317 (47.9)	<0.001	8,611 (40.5)	12,000 (47.8)	<0.001	14,578 (39.8)	19,317 (47.9)	<0.001

OAC, oral anticoagulants; SD, standard deviation; BMI, body mass index; GFR, glomerular filtration rate, calculated by CKDEPI; ACEI, angiotensin-converter enzyme inhibitors; ARB, angiotensin receptor blockers; NSAID, non-steroidal anti-inflammatory drugs.

## Variables

The variables assessed at baseline were: socio-demographic characteristics, toxic habits, comorbidities, body mass index (BMI), CHA_2_DS_2_VASc (score accounting for: C; congestive heart failure, H; hypertension, A_2_; age ≥75, D; diabetes, S_2_; prior stroke, V; vascular disease, A; age 65–75, Sc: sex category) (Lip et al., 2012), HAS-BLED (score accounting for Hypertension, Abnormal renal/liver function, Stroke, Bleeding history, Labile INR, Elderly, Drugs/alcohol) (Lip et al., 2011), and drug exposure to OAC.

### Drug exposure

Patients with NVAF were considered as exposed to any OAC if they started a new prescription during the study period (2011–2020). If they did not have any OAC prescription the prior 12 months, they were considered new users or naive patients. If they had received any other prescription of OAC the prior 12 months, different to the prescription that motivated the inclusion, they were defined as prevalent users or non-naive patients.

The drug exposure-related variables assessed after the DOAC treatment start were:

- Dose of DOAC; in order to ascertain the adequacy of dosing according to the Summary of Product Characteristics (SPC). Those people meeting these conditions but receiving the full dose were defined as “overdosed”, and those not meeting these conditions but receiving the reduced dose were defined as “underdosed”. For the conditions of dose reduction for each DOAC, please see Supplementary file, Supplementary Table S3.
- Treatment switch; when a different OAC is initiated during the study period.
- Discontinuation; defined as no dispensing of OAC during more than 2 months after having initiated treatment. Thus, persistence is defined as no discontinuation of OAC. Data included for discontinuation calculations: 2011–2019, as pharmacy invoice data might be registered after finishing the study period in December 2020.
- Adherence; measured by Medication Possession Ratio (MPR), which is the quotient between the drug amount dispensed and the drug amount prescribed during a defined period of time. MPR was calculated during all the time that a patient is receiving OAC treatment, thus, not discontinued it. A patient is adherent to the treatment when MPR ≥80%. Data included for adherence calculations: 2011–2019.

### Statistical analysis

The baseline characteristics of the cohort were described as relative and absolute frequencies for categorical variables and with mean and standard deviation (SD) or median and interquartile range (IQR) for quantitative variables. The results are shown for all the population included and stratified by gender. We performed a bivariate analysis across genders using the Chi-square test for categorical data and the Kaplan-Meier curves to estimate the restricted mean survival time for time to event variables.

The smooth algorithm was used to model the drug exposure. It is an automated method to obtain consistent patterns of drug exposition throughout study period (Ouchi et al., 2022). With the algorithm, we obtained the exact moment of each treatment presentation, combinations, additions, or interactions to other drugs, the time exposed to that treatment, moments of discontinuation, persistence and adherence.

We calculated adherence as an approximation of the medication possession ratio (MPR). It was the rate between the total days covered by the packages dispensed at the pharmacy by the total days of active prescriptions for that treatment. A threshold of 80% classified patients into adherents and non-adherents.

All statistical analyses were conducted with R software (version 4.1 or superior).

## Results

**FIGURE 1:**
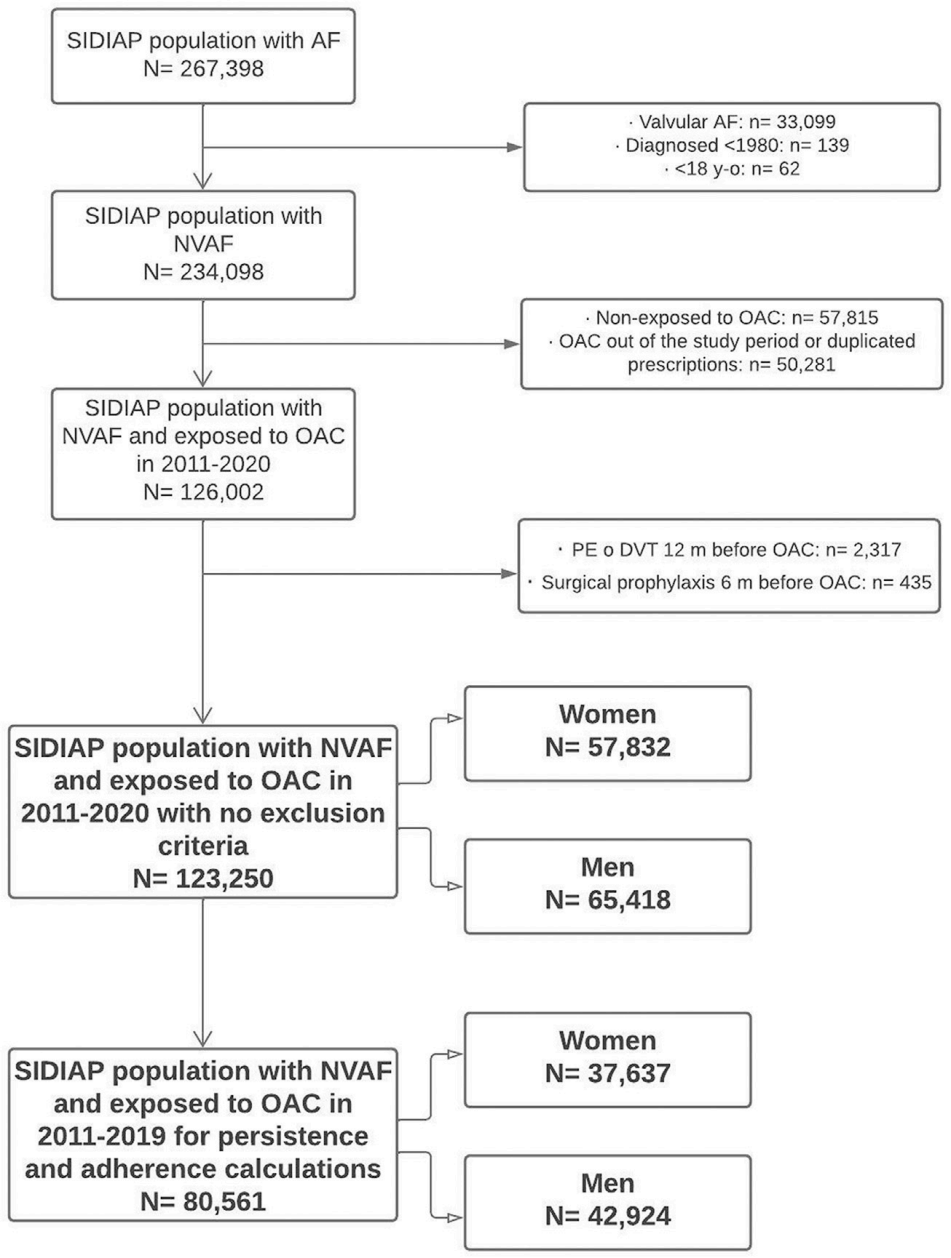
Flow diagram of population included. SIDIAP: Information System for Research in Primary Care. AF, atrial fibrillation; NVAF, non-valvular atrial fibrillation; OAC, oral anticoagulants; PE, pulmonary embolism; DVT, deep vein thrombosis.

During the study period, 123,250 people with NVAF initiated OAC treatment in our setting; 57,832 (46.9%) of them were women and 65,418 (53.1%) were men (Figure 1). Most patients (94.3% women and 94.5% men) were new users, and the OAC prescribed were VKA in 62.4% of patients (Table 1). The number of initiations with DOAC increased by year and decreased with VKA. Since 2019, DOAC accounted for more than 50% of treatment starts (Supplementary Table S4). Patients were followed-up during a mean of 45.6 months (SD 31.5); 45.7 months for women and 45.4 for men (*p* = 0.143).

Women were older than men, had higher BMI, and worse kidney function, whereas men were more frequently smokers and drinkers. HAS-BLED score was different between women and men; there were more men than women with scores 0–1 and more women than men with scores ≥2. Cancer, diabetes, ischaemic heart disease or previous stroke were more frequent in men, while dyslipidaemia or hypertension were more frequent in women. The most frequent comedications used were proton-pump inhibitors, beta-blockers, statins or angiotensin-converter enzyme inhibitors (ACEI), with differences between women and men for most drugs (Table 2).

**FIGURE 2:**
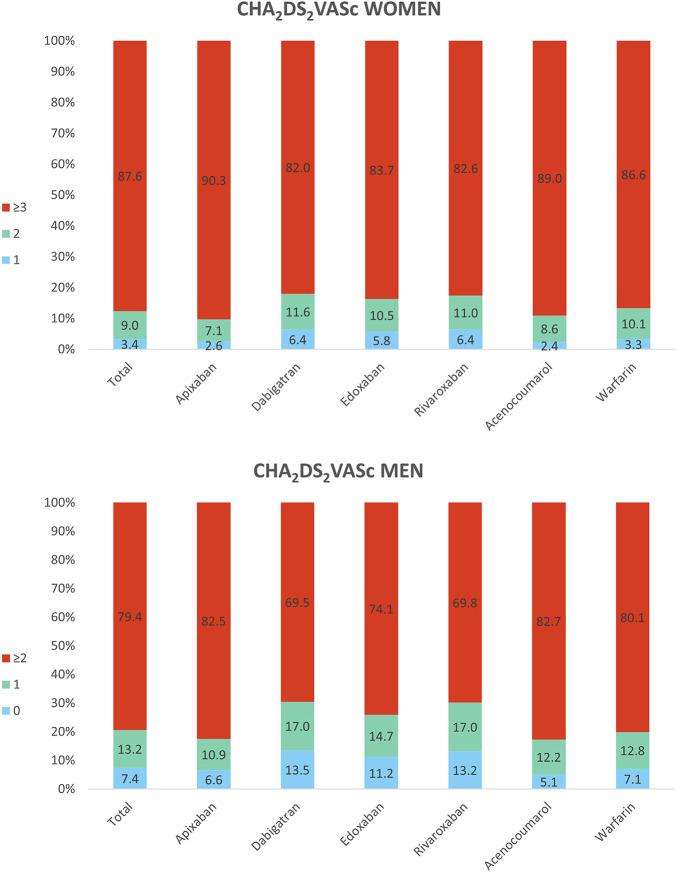
Baseline CHA_2_DS_2_VASc in women and men treated with oral anticoagulants. The top of Figure 2 shows the CHA_2_DS_2_VASc scores in all women receiving anticoagulants and by substance: 1, 2 and ≥3. The bottom shows the CHA_2_DS_2_VASc scores in all men receiving anticoagulants and by substance: 0, 1 and ≥2.

Regarding the stroke risk, women initiating DOAC had a mean CHA_2_DS_2_VASc score of 3.9 (SD 1.4) and men 2.6 (1.6), whereas it was 3.9 (1.2) in women and 2.7 (1.4) in men initiating VKA. In Figure 2 we see that 87.6% of anticoagulated women had a CHA_2_DS_2_VASc score ≥3, and there were 12.4% of women with a score ≤2 receiving OAC treatment, which would not be always indicated in this case. Apixaban showed the highest frequency of women with score ≥3 (90.3%) and dabigatran, the lowest (82%). As it is a sex-dependent measure, CHA_2_DS_2_VASc score was lower in men; 79.4% of men had CHA_2_DS_2_VASc ≥2 and 20.6%, <2. Also apixaban had the highest percentage of men with high stroke risk (82.5%) and dabigatran the lowest (69.5%).

**FIGURE 3:**
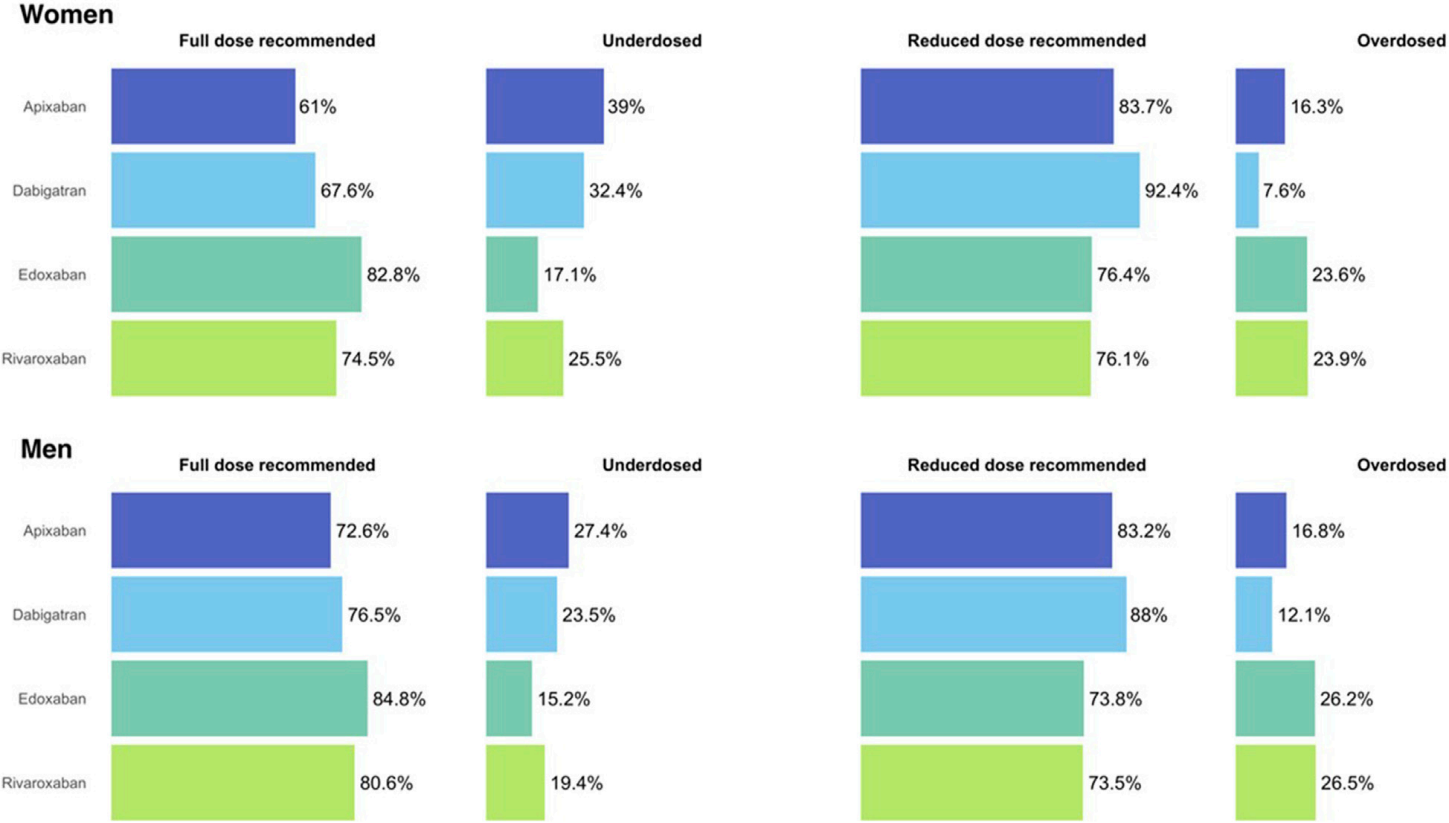
Dose adequacy in women and men initiating treatment with direct oral anticoagulants. Figure 3 shows the dose adequacy for all direct oral anticoagulants, women at the upper part and men at the bottom.

Regarding the dose adequacy to the SPC, women were more frequently underdosed than men for all DOAC, except for edoxaban (*p* = 0.355), being apixaban the DOAC with the highest frequency of underdosing; 39% of women and 27.4% of men (*p* < 0.001) were receiving a reduced dose despite they did not fulfil the criteria for the reduction. Men were more frequently overdosed than women, being significant only dabigatran (12% vs. 7.6%, *p* < 0.001). See Figure 3 and Supplementary Table S5.

There were 80,561 patients initiating OAC in 2011–2019 (Figure 1) and 28,840 of them initiated DOAC. The discontinuations and adherence to DOAC are shown in Table 3. Men discontinued DOAC treatment more frequently than women during the first year after initiation. Dabigatran showed the highest frequency of discontinuation (8.7% in women and 11.4% in men, *p* < 0.001). Rivaroxaban also did show differences between genders (8% vs. 9.2%, *p* = 0.038). Edoxaban demonstrated the lowest discontinuation rate (5.2% in women and 6.9% in men, *p* = 0.106), with differences in the time to discontinuation (4.5 months in women vs. 5.4 in men, *p* = 0.034). Discontinuations for apixaban were not different between genders (6.2% in women and 6.3% in men, *p* = 0.901). Regarding the time to discontinuation, we found differences between women and men for dabigatran (4.4 vs. 4.7 months, *p* = 0.014), but not for the rest of DOAC.

**TABLE 3:** Discontinuation and adherence to direct oral anticoagulants.

N (%)	Apixaban				Dabigatran				Edoxaban				Rivaroxaban			
All DOAC N = 28,840	Overall *n* = 10,184	Women *n* = 4,931 (48.4)	Men *n* = 5,253 (51.6)	*p*-value	Overall *n* = 6,123	Women *n* = 2,539 (41.5)	Men *n* = 3,584 (58.5)	*p*-value	Overall *n* = 3,233	Women *n* = 1,535 (47.5)	Men *n* = 1,698 (52.5)	*p*-value	Overall *n* = 9,300	Women *n* = 4,117 (44.3)	Men *n* = 5,183 (55.7)	*p*-value
1st year discontinuation	635 (6.2)	305 (6.2)	330 (6.3)	0.901	629 (10.3)	221 (8.7)	408 (11.4)	0.001	197 (6.1)	80 (5.2)	117 (6.9)	0.106	803 (8.6)	328 (8.0)	475 (9.2)	0.038
Months to discontinuation	—	—	—	0.695	—	—	—	0.014	—	—	—	0.324	—	—	—	0.155
Mean (SD)	5.0 (3.1)	5.0 (3.1)	5.0 (3.1)		4.6 (2.8)	4.4 (2.8)	4.7 (2.8)		5.0 (2.9)	4.5 (2.7)	5.4 (3.1)		4.6 (2.9)	4.5 (2.9)	4.7 (2.8)	
Median (IQR)	4.1 (2.3–7.2)	4.0 (2.3–7.0)	4.2 (2.3–7.4)		3.9 (2.2–6.5)	3.5 (1.9–6.4)	4.2 (2.4–6.5)		4.2 (2.7–6.9)	3.7 (2.6–6.1)	4.7 (3.1–7.6)		3.8 (2.2–6.3)	3.6 (2.0–5.9)	4.0 (2.3–6.5)	
MPR	—	—	—	0.142	—	—	—	0.104	—	—	—	0.708	—	—	—	0.006
≥80% (adherent)	8,451 (83.0)	4,124 (83.6)	4,327 (82.4)		5,257 (85.9)	2,202 (86.7)	3,055 (85.2)		2,745 (84.9)	1,309 (85.3)	1,436 (84.6)		7,875 (84.7)	3,532 (85.8)	4,343 (83.8)	
<80% (non-adherent)	1,733 (17.0)	807 (16.4)	926 (17.6)		866 (14.1)	337 (13.3)	529 (14.8)		488 (15.1)	585 (14.2)	262 (15.4)		1,425 (15.3)	585 (14.2)	840 (16.2)	

p-values for the comparison between genders. Chi-square test for categorical variables, and Kaplan-Meier curves to compare the restricted mean survival time to discontinuation (in months).

Adherence to all DOAC was high, as most patients had MPR ≥80%. Women and men showed differences for rivaroxaban (85.8% vs. 83.8%, *p* = 0.006). The other DOAC showed no differences; 83.6% in women and 82.4% in men for apixaban, 85.9% and 86.7% for dabigatran, and 84.9% and 85.3% for edoxaban.

## Discussion

We have analysed 10 years-data on OAC use for stroke prevention in NVAF in a PHC database in Catalonia, Spain. We found an increasing number of NVAF diagnoses over time, which obviously increased the number of people treated with OAC. Despite the criteria and recommendations in Spain positioning the VKA as the first choice in this indication up to the moment of data availability (Agencia Española de Medicamentos y Productos Sanitarios, 2012; Programa d’harmonització farmacoterapèutica, 2018), our study showed a progressive increase in the number of DOAC initiations and a decrease in VKA. In fact, the most updated information available indicates that DOAC account for more than 50% of the OAC initiations in NVAF.

There were more men in our study, as they are more frequently affected by AF (Hindricks et al., 2021). Comorbidities and risk of stroke and bleeding were similar to those described in prior investigations (Basaran et al., 2017; Giner-Soriano et al., 2020; Calderon et al., 2022), also with differences between men and women as previously described, as Loikas et al., who examined sex and gender differences in thromboprophylaxis in patients with NVAF. They reported more men with the disease but women with higher CHA_2_DS_2_VASc, as in our study. They also found that from 2011 to 2015 the number of women receiving OAC increased, except in older than 80, who were anticoagulated less frequently than in 2011 (Loikas et al., 2017).

We found that women were more frequently underdosed than men with all DOAC, even though they have a higher risk of stroke and the use of lower doses than recommended can presumably result in an increased rate of events (Raccah et al., 2018; Hindricks et al., 2021). We also found that men receiving dabigatran were more frequently overdosed than women, similar to the findings of Avgil Tsadok et al., where women were more frequently treated with dabigatran low dose than men (Avgil Tsadok et al., 2015). Carbone et al. found 29% of inadequate doses in octogenarian patients but being more frequently men those under- or overdosed (Carbone et al., 2022). Steinberg et al. found 13% of inadequate doses in elderly, women, and those with higher CHA_2_DS_2_VASc scores (Steinberg et al., 2016). In the past, we had already found a non-despicable frequency of inadequate doses for apixaban, dabigatran and rivaroxaban, although we had not studied gender differences (Programa d’harmonització farmacoterapèutica. et al., 2019). We believe that underdosing could have been caused by insufficient knowledge or lack of confidence in the appropriate dose (Sato et al., 2018; Cavillon Decaestecker et al., 2021), or by fear of the prescribers of causing harm, such as bleeding, as women present higher frequency and severity of adverse drug reactions than men (Tamargo et al., 2017; Fernández et al., 2020). We also think that prescribers might be more concerned for bleeding risk than for stroke risk, as overdosing was less frequent, and it might have been caused by insufficient knowledge of the criteria for dose reduction for each DOAC (Sato et al., 2018; Cavillon Decaestecker et al., 2021).

With regards to DOAC compliance, although studies used different methods to calculate persistence and adherence to treatment, we found better levels of persistence than other authors, close to 90% during the first year. For instance, Sabaté et al., with data proceeding from eight different databases in Europe, described persistence of 54–66% (Sabaté et al., 2021), or Banerjee et al.; who described persistence of 42.3–50.7%. Banerjee also assessed adherence levels calculating the Proportion of Days Covered (PDC), and approximately 60% of patients were considered adherents (Banerjee et al., 2020). In a comparable setting to ours, Hurtado-Navarro et al. found similar adherence levels to those in our study, also calculated by PDC, when they analysed those patients with at least two dispensings of the DOAC (Hurtado-Navarro et al., 2018). Although different levels of medication compliance have been described in women and men for different diseases (Billimek et al., 2015; Højgaard et al., 2018), none of the studies above analysed the differences in adherence and persistence to OAC treatment between women and men, and we did not find substantial differences between genders in our study.

The meta-analysis conducted by Raccah et al., in 2018 included the pivotal randomized clinical trials of the four DOAC with the purpose of evaluating whether the efficacy and safety of DOAC differed between genders, finding higher risk of stroke and lower risk of major bleeding in women in comparison with men (Raccah et al., 2018). They did not analyse doses prescribed, adherence or persistence.

Considering this lack of information on gender differences in NVAF and generally in medicine, and as sex and gender affect to all aspects of health and disease, it becomes necessary to carry out studies with gender perspective, allowing us to reduce health inequalities (McGregor et al., 2016; Madsen et al., 2017; Mauvais-Jarvis et al., 2020).

### Limitations and strengths

This study contains some limitations inherent to database studies, such as missingness for some variables, potential confounders or the lack of register of sex and gender variables and the consequent impossibility to know if they correspond to biological sex at birth or in the moment of register, together with the categorisation of this variable only as binary.

On the other hand, this study has some strengths as the large number of persons included, the representativeness of the general population, complete records, long follow-up periods, or real-world data (Recalde et al., 2022); apart from the fact of adding evidence to the pharmacological management of a very prevalent disease with a gender perspective.

## Conclusion

We analysed 10 years-data on OAC in our setting, describing the characteristics and gender differences of NVAF patients at treatment initiation.

We described the dose adequacy of patients receiving DOAC, finding a high frequency of underdosing, and significantly higher in women in comparison with men.

Adherence was generally high, only with higher levels in women for rivaroxaban. Persistence during the first year of treatment was also high in general, being more persistent women than men in the case of dabigatran and rivaroxaban.

## Study classification

Study classified by the Spanish Medicines Agency (AEMPS, *Agencia Española de Medicamentos y Productos Sanitarios*) as EPA-OD, code IDI-ACO-1019–01.

## Data Availability

The raw data supporting the conclusions of this article will be made available by the authors, without undue reservation.

## Abbreviations

ACEI, angiotensin-converter enzyme inhibitors; AF, atrial fibrillation; ARB, angiotensin receptor blockers; ATC, Anatomical, Therapeutic, Chemical classification system; BMI, body mass index; CMBD, database of diagnoses at hospital discharge; CHA2DS2VASc, Congestive heart failure, Hypertension, Age ≥75, Diabetes, Stroke, Vascular disease, Age 65–75, Sex category; DOAC, direct oral anticoagulants; DVT, deep vein thrombosis; ECAP, electronic health records in Primary Health Care in Catalonia; HAS-BLED, Hypertension, Abnormal renal/liver function, Stroke, Bleeding history, Labile INR, Elderly, Drugs/alcohol; ICD, International Classification of Diseases; INR, International Normalized Ratio; IQR, interquartile range; LTCF, long-term care facilities; MPR, medication possession ratio; NSAID, non-steroidal anti-inflammatory drugs; NVAF, non-valvular atrial fibrillation; OAC, oral anticoagulants; PE, pulmonary embolism; PHC, Primary Health Care; SD, standard deviation; SIDIAP, Information System for Research in Primary Care; SPC, summary of product characteristics; TTR, time in therapeutic range; VKA, vitamin K antagonists.
