# Supplementary material for: Sex and gender differences in the use of oral anticoagulants for non-valvular atrial fibrillation: A population-based cohort study in primary health care in catalonia
Source: Front Pharmacol. 2023 Feb 7;14:1110036. doi: 10.3389/fphar.2023.1110036 (PMC9941166; doi:10.3389/fphar.2023.1110036)
Supplement: Supplementary file 1 [file DataSheet1.docx]

# Supplementary file

## Supplementary table 1. ICD-10 codes for diagnoses and comorbidities

| **DIAGNOSES** | **ICD-10 CODES** |
| --- | --- |
| **INCLUSION CRITERIA** | |
| Atrial fibrillation | I48 |
| **EXCLUSION CRITERIA** | |
| Deep vein thrombosis | I82 |
| Pulmonary embolism | I26 |
| Valvular disease | I05, I08 |
| Surgical prophylaxis, hip replacement | 0S2, 0SB9, 0SBB, 0SC9, 0SCB, 0SH9, 0SHB, 0SQ9, 0SQB, 0SR9, 0SRA, 0SRB, 0SRE, 0SRR, 0SRS, 0SS9, 0SSB, 0ST9, 0STB, 0SW9, 0SWA, 0SWB, 0SWE, 0SWR, 0SWS |
| Surgical prophylaxis, knee replacement | 0S2, 0SBC, 0SBD, 0SCC, 0SCD, 0SHC, 0SHD, 0SQC, 0SQD, 0SRC, 0SRD, 0SRT, 0SRU, 0SRV, 0SRW, 0SSC, 0SSD, 0STC, 0STD, 0SWC, 0SWD, 0SWT, 0SWU, 0SWV |
| **COMORBIDITIES** | |
| Cancer | C00-C97 |
| Chronic kidney disease | N18 |
| Diabetes mellitus | E10-E14 |
| Dislypidaemia | E78 |
| Gastrointestinal haemorrhage | K25.0, K25.2, K25.4, K25.6, K26.0, K26.2, K26.4, K26.6, K27.0, K27.2, K27.4, K27.6, K28.0, K28.2, K28.4, K28.6, K29.0, K29.6, K29.7 |
| Heart failure | I50 |
| Hypertension | I10-I15 |
| Intracranial haemorrhage | I61-I62 |
| Ischaemic heart disease | I20-I25 |
| Liver disease | K70-K77 |
| Peripheral artery disease | I70, I73, I74 |
| Stroke | I63-I69 |
| Subaracnoidal haemorrhage | I60 |
| Transient ischaemic attack | G45 |

## Supplementary table 2. ATC codes for drugs of interest and comedications

| **DRUGS OF STUDY** | |
| --- | --- |
| Acenocoumarol | B01AA07 |
| Apixaban | B01AF02 |
| Dabigatran | B01AE07 |
| Edoxaban | B01AF03 |
| Rivaroxaban | B01AF01 |
| Warfarin | B01AA03 |
| **COMEDICATIONS** | |
| Angiotensin converting enzyme inhibitors | C09A, C09B |
| Angiotensin II receptor blockers | C09C, C09D |
| Antiarrhythmics and digoxin | C01A, C01B |
| Antihypertensive drugs | C02 |
| Antiplatelets | B01AC |
| Beta blockers | C07 |
| Calcium antagonists | C08CA, C08D |
| Diuretics | C03 |
| Drugs for diabetes | A10 |
| Nitrates | C01DA |
| Non-steroidal anti-inflammatory drugs | M01A, N02BA, N02BB |
| Other anticoagulants | B01AB, B01AD, B01AX |
| Proton pump inhibitors | A02BC |
| Statins and lipid modifying agents | C10 |
| Systemic corticosteroids | H02 |

## Supplementary table 3. Conditions for dose reduction of direct oral anticoagulants according to the Summary of Product Characteristics

| **Drug** | **Full dose recommended** | **Dose reduction conditions** |
| --- | --- | --- |
| **Apixaban** | **5 mg/12h** | **2.5 mg/12h** when at least one of the following criteria is met at the treatment start:  - 2 of 3: i) serum creatinine ≥ 1.5 mg/dL; ii) age ≥ 80; iii) weight ≤ 60 kg  - Glomerular filtration rate (GFR) < 30 mL/min (not recommended if < 15 mL/min) |
| **Dabigatran**  *75 mg dose is not authorized for non-valvular atrial fibrillation (NVAF) | **150 mg/12h** | **110 mg/12h** when any of the following criteria is met at the treatment start:  - Age ≥ 80  - GFR = 30-50 mL/min (contraindicated if < 30 mL/min)  - Simultaneous treatment with verapamil |
| **Edoxaban**  *15 mg dose is not authorized for NVAF | **60 mg/24h** | **30 mg/24h** when any of the following criteria is met at the treatment start:  - GFR = 15-50 mL/min (contraindicated if < 15 mL/min)  - Weight ≤ 60 kg  - Treatment with glycoprotein P inhibitors (cyclosporine, dronedarone, erythromycin) |
| **Rivaroxaban**  *10 mg dose is not authorized for NVAF | **20 mg/24h** | **15 mg/24h** if GFR < 50 mL/min at the treatment start (contraindicated in < 15 mL/min). |

## Supplementary table 4. Oral anticoagulant initiations per year during 2011-2020

In this graphical representation we see that the number of treatment initiations with direct oral anticoagulants (DOAC: apixaban, dabigatran, edoxaban and rivaroxaban) increased by year during the study period and decreased the number of initiations with vitamin K antagonists (acenocoumarol and warfarin). Since 2019, DOAC accounted for more than 50% of treatment starts.

*Percentages calculated by row.

## Supplementary table 5. Dose adequacy to the Summary of Product Characteristics in the population treated with direct oral anticoagulants

In this table we present the results of the Chi-square test comparing distribution between women and men for overdosed and underdosed, according to the conditions for prescribing full dose or reduced dose explained in Supplementary table 1.

|  |  | **Overall** | **Women** | **Men** | **p-value*** |
| --- | --- | --- | --- | --- | --- |
| **APIXABAN** | **N** | 1654 | 933 | 721 |  |
|  | **reduced recommended** | 1381 (83.5) | 781 (83.7) | 600 (83.2) | 0.842 |
|  | **overdosed** | 273 (16.5) | 152 (16.3) | 121 (16.8) |  |
|  | **N** | 11701 | 5691 | 6010 |  |
|  | **underdosed** | 3865 (33.0) | 2220 (39.0) | 1645 (27.4) | <0.001 |
|  | **full recommended** | 7836 (67.0) | 3471 (61.0) | 4365 (72.6) |  |
| **DABIGATRAN** | **N** | 3015 | 1637 | 1378 |  |
|  | **reduced recommended** | 2724 (90.3) | 1512 (92.4) | 1212 (88.0) | <0.001 |
|  | **overdosed** | 291 (9.7) | 125 (7.6) | 166 (12.0) |  |
|  | **N** | 5142 | 1824 | 3318 |  |
|  | **underdosed** | 1371 (26.7) | 591 (32.4) | 780 (23.5) | <0.001 |
|  | **full recommended** | 3771 (73.3) | 1233 (67.6) | 2538 (76.5) |  |
| **EDOXABAN** | **N** | 995 | 644 | 351 |  |
|  | **reduced recommended** | 751 (75.5) | 492 (76.4) | 259 (73.8) | 0.403 |
|  | **overdosed** | 244 (24.5) | 152 (23.6) | 92 (26.2) |  |
|  | **N** | 1444 | 554 | 890 |  |
|  | **underdosed** | 230 (15.9) | 95 (17.1) | 135 (15.2) | 0.355 |
|  | **full recommended** | 1214 (84.1) | 459 (82.9) | 755 (84.8) |  |
| **RIVAROXABAN** | **N** | 1377 | 765 | 612 |  |
|  | **reduced recommended** | 1032 (74.9) | 582 (76.1) | 450 (73.5) | 0.307 |
|  | **overdosed** | 345 (25.1) | 183 (23.9) | 162 (26.5) |  |
|  | **N** | 9660 | 4197 | 5463 |  |
|  | **underdosed** | 2132 (22.1) | 1071 (25.5) | 1061 (19.4) | <0.001 |
|  | **full recommended** | 7528 (77.9) | 3126 (74.5) | 4402 (80.6) |  |
